# Diagnostic efficacy and value of ultrasound in children’s scrotal testicular torsion: A retrospective analysis

**DOI:** 10.1097/MD.0000000000039884

**Published:** 2024-10-11

**Authors:** Chen Zeng, Lixiang Fang, Wei Li, Hailan Chen

**Affiliations:** a Department of Ultrasound, Putian University Affiliated Hospital, Putian, Fujian, China.

**Keywords:** manual reduction, orchiectomy, testicular survival rate, testicular torsion, ultrasound

## Abstract

The purpose of this study is to explore the diagnostic efficacy and value of ultrasound detection for testicular torsion in children with scrotal and testicular diseases. A total of 120 children with acute scrotal swelling and pain who were treated in our hospital from August 2017 to August 2022 were selected for preliminary diagnosis through color Doppler ultrasound diagnostic instrument examination. The final diagnosis was made through surgical or conservative treatment. At the same time, 40 children with acute epididymitis during the same period were selected as the control group, and the clinical treatment of patients with testicular diseases was retrospectively analyzed. A total of 120 children were diagnosed with testicular torsion disease, with 57 cases affecting the left testicle and 63 cases affecting the right testicle. Ultrasound examinations revealed no blood flow signal in 78 cases, a significant reduction in blood flow in 38 cases, and no change in 4 cases. Among the pediatric patients who underwent manual reduction, 79 cases had a favorable prognosis. Surgical reduction was performed in 41 cases, with 35 cases successfully treated and 6 cases resulting in testicular removal. Follow-up examinations conducted 6 months to 1 year postoperatively showed testicular atrophy in 4 out of 35 cases with preserved testicles, while the 6 cases that underwent testicular resection had good outcomes. The non-active subgroup had a longer disease course and a greater degree of torsion (*P* < .05). There was no statistically significant difference in testicular volume and the ratio of healthy testicular volume between the 2 groups (*P* > .05). The sensitivity of ultrasound diagnosis was 95.24% (73/77), specificity was 78.57% (34/43), and accuracy was 89.29% (107/120). Ultrasound can effectively diagnose testicular torsion and evaluate the success rate of testicular reduction. Early treatment of patients with testicular torsion leads to better efficacy and higher survival rates.

## 1. Introduction

Testicular torsion, or spermatic cord torsion, is a prevalent urological emergency primarily affecting adolescents and children.^[1–3]^ Misdiagnosis, often due to symptom overlap with acute epididymitis or orchitis, can lead to severe outcomes such as testicular necrosis or irreversible atrophy if treatment is delayed.^[4–6]^ Previous studies in this region, have highlighted issues with delayed diagnosis and treatment, emphasizing the need for improved diagnostic and therapeutic protocols. These studies reported a significant incidence of misdiagnosis and suboptimal treatment outcomes, which underscores the critical need for more effective management strategies.

Given these findings, the current study aims to address these gaps by conducting a comprehensive retrospective analysis of 120 cases of testicular torsion diagnosed and treated at our hospital between August 2019 and August 2022. The study focuses on evaluating the diagnostic and treatment processes, identifying patterns of misdiagnosis, and assessing the effectiveness of postoperative ultrasound evaluations in improving patient outcomes. By enhancing our understanding of these processes, the study seeks to contribute to reducing diagnostic errors and improving the standard of care for testicular torsion in this region.

## 2. Data and methods

### 2.1. Clinical data

This study was approved by the Ethics Committee of Putian University Affiliated Hospital. A total of 160 patients under the age of 12 were included in this study, conducted from August 2017 to August 2022. Among them, 57 patients had left testicular torsion and 63 had right testicular torsion. The time from onset to visit varied, with 16 cases presenting within 0 to <6 hours, 38 cases within 6 to <12 hours, 38 cases within 12 to <48 hours, and 10 cases beyond 48 hours. Due to some commonality of testicular torsion symptoms, it is easy to be misdiagnosed as acute epididymitis or delayed orchitis, resulting in testicular necrosis or irreversible testicular atrophy. Therefore, children with acute epididymitis were selected as the control group, and the 2 groups were compared and analyzed. As a control group, 40 children with acute epididymitis, aged <12 years and diagnosed through epididymostomy, were selected. Inclusion criteria required patients to be younger than 12 years old, examined by a urologist and ultrasound specialist before surgery, confirmed with testicular torsion through surgery, and followed up for at least 6 months postoperatively. Exclusion criteria included severe renal, liver, and heart dysfunction, as well as incomplete medical records and follow-up information. The study adhered to the Helsinki Declaration and obtained informed consent from all patients.

### 2.2. Diagnosis and treatment

#### 2.2.1. Ultrasound examination

The GE Logiq E9 and GE LOGIQ E11 color ultrasonic diagnostic instrument was utilized for the study, specifically using the linear array probe with a frequency range of 7.5 to 12.0 MHz. The two-dimensional ultrasound scan was performed to observe the size, echo, blood flow, and fluid accumulation in the vaginal cavity of the testis. Additionally, the conditions of the epididymis were also examined.^[7]^The testicular volume ratio (C = twisted side/healthy side) can be calculated by using 0.71 × long × wide × thickness to measure the bilateral testicular diameter through ultrasound. The bleeding severity is classified into 3 levels according to the Arada scoring system: Level I indicates significant and rapid bleeding, Grade II indicates bleeding within 10 minutes, and Grade III indicates no bleeding within 10 minutes. Based on the Arada score, 120 children with testicular torsion were divided into 2 subgroups: the first and second grade subgroups with testicular vitality underwent testicular reduction, while the third grade subgroup with testicular inactivity underwent surgery to remove necrotic testicles or fix the affected testicles. Intraoperative testing was used to further divide the 120 children with testicular torsion into subgroups ≤360° and subgroups >360° based on the degree of testicular torsion. Additionally, based on the duration of symptoms, the 120 children with testicular torsion were divided into subgroups ≤24 hours and subgroups >24 hours.

#### 2.2.2. Treatment methods

The time from symptom onset to hospital visit ranged from 0 to 6 hours. The patient underwent manual reduction treatment, followed by color Doppler ultrasonography to observe the blood flow of the affected testis. Surgical exploration was conducted within 6 to 24 hours after symptom onset. The surgical procedure involved making an incision in the scrotum to expose the testicular vaginalis, spermatic cord, and epididymis. The twisted testis was then reset, and warm water was applied to the spermatic cord for 30 minutes to promote blood flow recovery. The testis exhibited a change in color to red, with restored blood vessel pulsation, after which testis fixation was performed. If there was no change in testicle color and blood vessel pulsation, the white testicle membrane was incised and if no blood oozed out, testicle excision was performed. Regardless of the reduction method used, ultrasound was performed after reduction to evaluate the restoration of testicular blood supply.

#### 2.2.3. Observing indicators

Intratesticular blood flow information was evaluated using color Doppler ultrasound (CDUS). The changes in blood flow were assessed to determine the success of reduction. A gradual increase in blood flow indicates a successful reduction, while the absence of an increase in blood flow signal suggests a failed reduction.

#### 2.2.4. Statistical analysis

The data were processed and analyzed by SPSS 22.0. According to the data characteristics of clinical factors, the χ^2^ test method was selected to deal with the data relationship, *P* < .05 as the significant difference.

## 3. Results

### 3.1. Ultrasonic diagnosis

**Figure 1.**
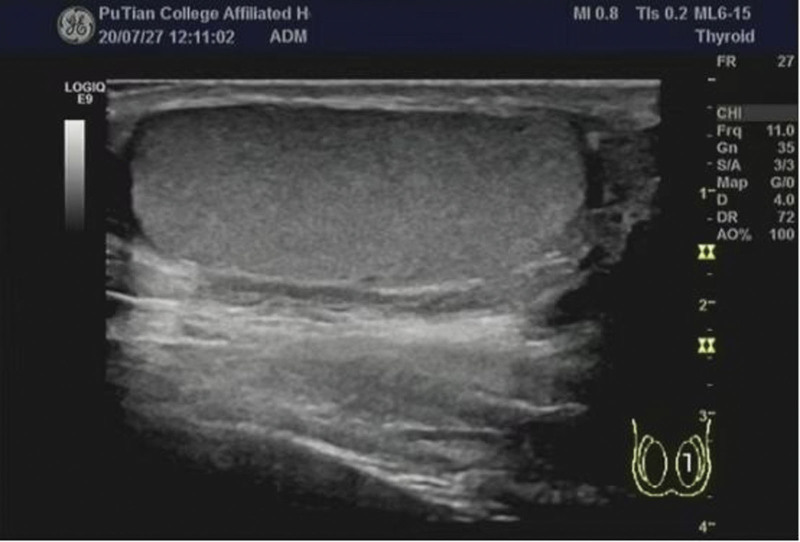
Longitudinal section of testis.

**Figure 2.**
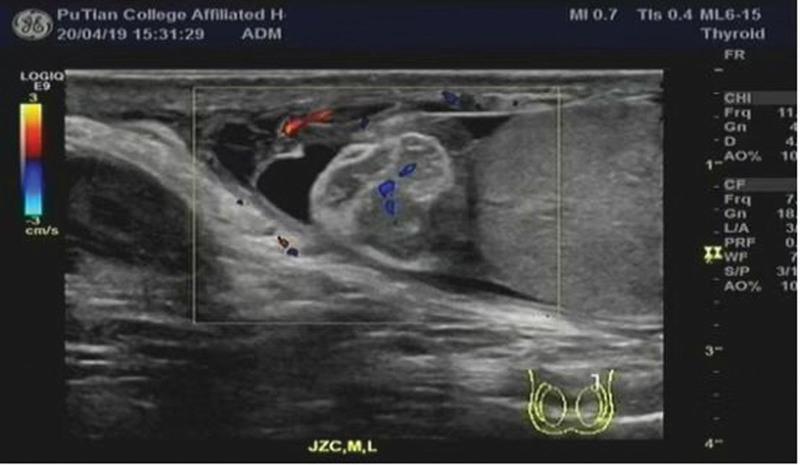
Uneven echo clumps of epididymal head above testis.

**Figure 3.**
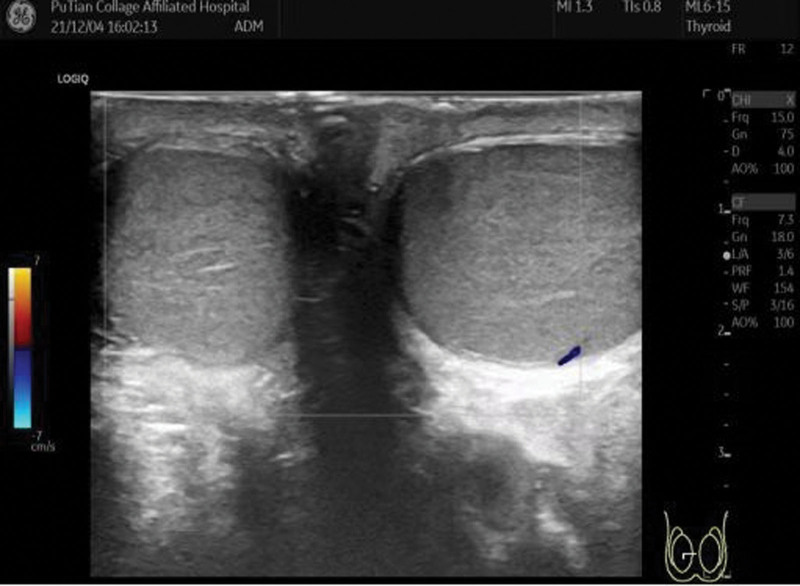
Doppler bilateral comparison.

Among the 120 patients studied, 57 were found to have testicular torsion on the left side and 63 on the right side. Two-dimensional ultrasound examinations revealed varying degrees of enlargement in the affected testicles, particularly in the anterior–posterior diameter, resulting in a nearly spherical shape (Fig. 1). At times, the testicles exhibited abnormal transverse positions. Echogenicity was either uniform or unevenly reduced, often accompanied by necrosis or liquefaction, with visible punctate or patchy areas of strong echogenicity alongside regions of low to no echogenicity. In 109 cases with torsion and necrosis of the epididymis and spermatic cord, isolated masses of uneven strong echogenicity were observed above or below the testicles. The affected side of the scrotum frequently displayed small anechoic dark areas, along with significant thickening of the scrotal wall (Fig. 2). Doppler energy imaging showed no blood flow signal in 78 cases, significant reduction in testicular blood flow in 38 cases, and no change in testicular blood flow in 4 cases (Fig. 3). Manual reduction was performed in 79 cases, with 79 cases undergoing ultrasound examination postreduction to ensure normal blood supply to the testicles. Surgical reduction was carried out in 41 cases, with 35 cases showing normal blood flow post-ultrasound evaluation, while 6 cases necessitated testicular resection after failed reduction attempts.

### 3.2. Ultrasound parameters of testicles in pediatric patients

Compared to the control group, the testicular torsion group showed a statistically significant difference in Arda scores (*P* < .05). Both groups had a younger age, and there was no significant difference in disease duration, testicular volume on the affected and healthy sides, and volume ratio on the affected and healthy sides (*P* > .05), as presented in Table 1.

**Table 1:** Comparison of testicular ultrasound parameters between the observation group and the control group.

Parameter	Testicular torsion group (120 cases)	Control group (40 cases)	t/χ^2^	*P*
Age	3.97 ± 1.55	3.41 ± 1.01	2.000	.052
course (h)	49.05 ± 15.16	50.11 ± 16.22	0.251	.804
Arda rating			16.920	.000
I,II	80	40		
III	40	0		
Affected testicular volume (mL)	2.75 ± 1.02	2.49 ± 1.11	1.187	.242
Healthy testicular volume (mL)	1.56 ± 1.04	1.52 ± 0.96	0.192	.849
Affected healthy side volume ratio	1.75 ± 0.97	1.64 ± 0.99	0.543	.590

### 3.3. Comparison of testicular ultrasound parameters in children with different Arda score subgroups

Compared to the active subgroup, the non-active subgroup had a longer disease course and a greater degree of torsion (*P* < .05). There was no statistically significant difference in testicular volume and the ratio of healthy testicular volume between the 2 groups (*P* > .05), as shown in Table 2.

**Table 2:** Comparison of testicular ultrasound parameters in children with different Arda score subgroups.

Parameter	≤360° subgroup/(55 cases)	>360° subgroup/(65 cases)	t/χ^2^	*P*
Age	5.03 ± 1.97	1.92 ± 1.27	6.233	<.001
Course (h)	30.05 ± 10.09	59.61 ± 20.37	7.293	<.001
Affected testicular volume (mL)	2.95 ± 1.12	2.36 ± 0.86	2.007	.060
Healthy testicular volume (mL)	1.74 ± 1.09	1.21 ± 0.95	1.796	.089
Affected healthy side volume ratio	1.70 ± 1.08	1.95 ± 0.78	0.895	.383
Torsion degree (°)	532.78 ± 128.80	727.50 ± 178.59	4.684	<.001

### 3.4. Comparison of testicular ultrasound parameters in subgroups of children with different degrees of torsion

Compared to the subgroup with angles ≤360°, the subgroup with angles >360° had a younger age, longer disease course, and higher Arda score (*P* < .05). There was no statistically significant difference in testicular volume and healthy side volume ratio between the 2 subgroups (*P* > .05), as shown in Table 3.

**Table 3:** Comparison of testicular ultrasound parameters in different subgroups.

Parameter	Active subgroup/(76 cases)	Inactive subgroup/(44 cases)	t/χ^2^	*P*
Age	4.81 ± 1.68	3.50 ± 1.48	3.016	.005
Course (h)	39.06 ± 12.15	54.61 ± 20.41	5.906	.000
Arda rating			6.162	.013
I,II	51	16		
III	25	28		
Affected testicular volume (mL)	2.62 ± 0.99	2.82 ± 1.08	0.691	.494
Healthy testicular volume (mL)	1.52 ± 0.98	1.58 ± 1.08	0.213	.832
Affected healthy side volume ratio	1.70 ± 1.08	1.76 ± 1.01	0.170	.866

### 3.5. Treatment

A total of 120 cases of testicular torsion were observed in this study. Among them, 69 cases were identified in the early stage and were successfully treated through manual reduction, resulting in a good prognosis. For cases in the middle stage, surgical reduction and fixation of the testicle were performed in 31 patients. However, in 6 cases, the reduction was unsuccessful, leading to the need for testicle resection. In 20 cases of advanced patients, surgical resection was necessary due to necrotic testicular tissue. After a follow-up period of 6 months to 1 year, it was observed that 4 out of 26 cases with preserved testis experienced atrophy. On the other hand, the follow-up of 22 patients who underwent testicectomy showed positive outcomes.

### 3.6. Clinical efficacy of testicular torsion in children

A total of 120 cases of testicular torsion were diagnosed using ultrasound, while 73 cases of testicular torsion in children were diagnosed by ultrasound, and 77 cases were clinically and pathologically diagnosed. Using clinical and pathological results as the gold standard, the sensitivity of ultrasound diagnosis was 95.24% (73/77), specificity was 78.57% (34/43), and accuracy was 89.29% (107/120), as shown in Table 4.

**Table 4:** Results of ultrasonic diagnosis of testicular torsion in children (cases).

Ultrasonic diagnosis	Clinicopathology		Total
	Positive	Negative	
Positive	73	9	82
Negative	4	34	38
Total	77	43	120

## 4. Discussion

Testicular torsion is a common emergency disease in urology. Its signs include testicular pain with swelling, spermatic cord thickening, testicular displacement, nausea, and vomiting. In most patients, the scrotal elevation test is positive, and the cremaster reflex is absent. During acute attacks, testicular pain is severe, and it is easy to misdiagnose diseases such as acute inflammation of the testis or epididymis, torsion of testicle appendices, and incarcerated hernia.^[8,9]^ Testicular torsion occurs when the blood circulation in the spermatic cord is blocked, leading to testicular ischemia and necrosis. The survival rate of the testicle is closely related to the degree of torsion and the duration of the disease.^[10]^ Experimental studies have shown that irreversible damage occurs within 2 hours after the onset of testicular torsion, and complete infarction occurs before 24 hours. Therefore, early diagnosis and timely and correct treatment can significantly reduce the testicular resection rate.^[11]^ Ultrasound is a simple method of choice for diagnosing testicular torsion. It can detect abnormal testicular essence echo, decreased testicular internal blood flow, or even its disappearance.^[10]^

In this study group, 96 cases of testicular enlargement due to affected testicles were diagnosed using ultrasound, while 24 cases were not. Among the diagnosed cases, 78 showed a disappearance of testicular blood flow signal, 38 had a significant decrease in testicular blood flow (compared to the healthy side), and 4 cases showed no change in testicular blood flow. Additionally, 109 cases exhibited reduced echo, while 11 cases had mixed echo. The decrease in testicular blood flow is not significant, and there is an approximately normal blood flow signal. This signal is related to the immature development of the testicles in infants and young children, as well as low blood flow and thickness of the spermatic cord. It is challenging to deny testicular torsion solely based on the presence of blood flow signals in the testicles. The thicker the spermatic cord, the longer the torsion, and the ultrasound of the twisted spermatic cord can display a snail shell-like appearance. This is usually considered a sensitive or specific sign of testicular torsion and may have even more diagnostic value than abnormal testicular blood flow. However, in cases where the degree of testicular torsion is small and the torsion time is short, there may be no significant change in arterial blood flow due to blocked venous reflux. This can lead to false positive or negative results. For instance, in this study, color Doppler flow imaging testing of individual children with testicular torsion showed widening of the spermatic vein and the detection of red and blue blood flow signals. Intermittent testicular torsion can occur, where the symptoms persist for a long time but the actual time of testicular ischemia is short. In such cases, similar normal blood flow signals may also appear, leading to misjudgment.

In this study, it was found that the rate of inactivation was higher for testicles that were stored for more than 24 hours compared to those stored for 24 hours or less. Additionally, the rate of inactivation was higher for testicles with a torsion degree >360° compared to those with a torsion degree of 360° or less. This suggests that the development of ischemic necrosis due to testicular torsion is influenced by both the angle and duration of torsion. Testicular necrosis occurs 3 to 4 days after torsion when the torsion is 180°, 12 to 24 hours after torsion when the torsion is 360°, and 2 hours after torsion when the torsion is 720°. The severity of damage to the blood circulation of the testes increases with the degree of torsion.^[9]^ Inactive testes typically exhibit longer symptom duration, and testicular torsion >360° is associated with a higher likelihood of testicular loss. Cuckow et al^[12]^ proposed that testicular torsion >360° and duration longer than 24 hours may require testicular resection. In this study, among the children with torsion lasting more than 24 hours, 11 still had testicular activity, and among those with torsion >360°, 19 still had testicular activity. This can be attributed to 2 reasons: ① some testicular torsion did not exceed 360°, resulting in blocked venous return but normal blood supply from the testicular artery, preventing testicular ischemic death; ② the same degree of torsion can have varying levels of tension and impact on testicular blood supply.^[13,14]^

Testicular torsion and acute epididymitis are common emergencies in pediatric urology, characterized by sudden red swelling and pain of the scrotum, accompanied by tenderness or edema. The clinical manifestations of these conditions are similar and difficult to distinguish. In this study, out of the 120 cases of testicular torsion, 3 cases were misdiagnosed as acute epididymitis. The misdiagnosis may be attributed to the early stage of torsion, where the increased peak arterial flow velocity compensates for testicular ischemia, resulting in no change in the blood flow signal of the affected testicle and an increase in the normal testicular blood flow signal, giving a false appearance of inflammatory lesions. The ultrasound findings of acute epididymitis in this study were typical, with rich blood flow in the testicles and/or epididymis, uniform internal echoes, no thickening of the spermatic cord, and clear dissection. When children lie on their backs, gently raising the testicles can relieve pain by reducing the tension of the spermatic cord, but in children with testicular torsion, the pain worsens. Furthermore, in cases where the testicles of children are undeveloped and show very little or no blood flow signal on CDUS, diagnosing testicular torsion by comparing blood flow changes may also lead to misdiagnosis. To improve accuracy, it is recommended to start the ultrasound examination from the healthy side of the testicles, optimize CDUS parameters, and then examine the affected side with the same settings. Comparing the transverse sections of the 2 testicles can provide transverse color static images of the midline, allowing for a comparison of the blood flow signals. If there is a reduction in blood flow signals on one side, it should raise a high suspicion of testicular torsion.

For early testicular torsion (<6 hours) without scrotal skin and spermatic cord edema, the first step is manual reduction. It is important not to administer analgesics during the reduction process as they may interfere with pain assessment and the success of reduction. During the reduction, it is crucial to pay attention to rotating in the direction that provides pain relief. The patient should be asked to provide feedback, allowing for adjustments in reduction strength and amplitude. After successful manual reduction, orchiopexy should be performed to prevent future occurrences of testicular torsion. In cases where manual reduction fails, prompt surgical reduction is necessary.^[15]^A total of 79 cases of early testicular torsion in this group were managed with manual reduction, resulting in a favorable prognosis. Surgical exploration and reduction are recommended for patients with testicular torsion lasting over 6 hours. Timely surgical resection is indicated for those with confirmed testicular damage. The success of manual reduction depends on the timing and morphology of the torsion. Among the cases that underwent surgical reduction, 35 were successful with normal blood flow post-surgery, as confirmed by ultrasound. However, 6 cases required testicular resection due to failed reduction. Regardless of the reduction method used, ultrasound evaluation is essential to assess the recovery of testicular blood supply. During a follow-up period of 6 months to 1 year postoperatively, 4 out of 35 cases with preserved testicles exhibited testicular atrophy on the affected side, while the 6 cases that underwent testicular resection showed favorable outcomes during follow-up.

Relevant studies have found that the diagnostic sensitivity of CDUS for testicular torsion can reach 80% to 98%, and the specificity can reach 97%.^[16]^ However, the clinical features of acute orchitis and testicular torsion are similar, such as sudden swelling and pain of the scrotum, with or without tenderness and edema, making it difficult to distinguish the ultrasonography. The results of this study showed that 6 cases were misdiagnosed by clinicopathology. The sensitivity of ultrasound diagnosis was 95.24% (120/126), the specificity was 78.57% (55/70), and the accuracy was 89.29% (175/196). The reasons for misdiagnosis may be: the increase of arterial flow rate in early torsion compensated for testicular ischemia, resulting in no change in blood flow signal of the affected testicle, but increased blood flow signal of the normal testicle, resulting in the illusion of inflammatory lesions.^[17]^ This study still has limitations. It is a retrospective study with a small sample size of patients, which does not affect the accuracy and credibility of this study, but may not be so convincing. Meanwhile, we did not include the comparison between Doppler ultrasound parameters and disease conditions.

Testicular torsion is a common urinary emergency in adolescence, with spermatic cord torsion being the most prevalent. This condition can cause a decrease in testicular blood flow, leading to potential damage or loss of the gonads if left untreated. Ultrasound is an effective diagnostic tool for testicular torsion and can also assess the success rate of testicular reduction. Early diagnosis and treatment are crucial in managing testicular torsion, as they can significantly improve the chances of survival.^[18,19]^

## Author contributions

**Conceptualization:** Chen Zeng, Wei Li, Hailan Chen.

**Data curation:** Lixiang Fang, Hailan Chen.

**Formal analysis:** Chen Zeng, Hailan Chen.

**Funding acquisition:** Chen Zeng, Wei Li.

**Investigation:** Chen Zeng, Lixiang Fang, Wei Li, Hailan Chen.

**Methodology:** Chen Zeng, Lixiang Fang, Wei Li, Hailan Chen.

**Validation:** Lixiang Fang, Wei Li.

**Writing – original draft:** Chen Zeng.

**Writing – review & editing:** Chen Zeng.

## References

[1] Kumar M Gautam V. Testicular torsion. N Engl J Med. 2021;385:1603. 34652889 10.1056/NEJMicm2110702

[2] Keays M Rosenberg H. Testicular torsion. CMAJ. 2019;191:E792. 31308008 10.1503/cmaj.190158 PMC6629539

[3] Shunmugam M Goldman RD. Testicular torsion in children. Can Fam Physician. 2021;67:669–71. 34521708 10.46747/cfp.6709669 PMC9683365

[4] Ta A D’Arcy FT Hoag N D’Arcy JP Lawrentschuk N. Testicular torsion and the acute scrotum: current emergency management. Eur J Emerg Med. 2016;23:160–5. 26267075 10.1097/MEJ.0000000000000303

[5] Sharp VJ Kieran K Arlen AM. Testicular torsion: diagnosis, evaluation, and management. Am Fam Physician. 2013;88:835–40. 24364548

[6] Hu S Guo M Xiao Y. Mapping trends and hotspot regarding testicular torsion: A bibliometric analysis of global research (2000-2022). Front Pediatr. 2023;11:1121677. 36925671 10.3389/fped.2023.1121677 PMC10011162

[7] Hosokawa T Tanami Y Sato Y Ishimaru T Kawashima H Oguma E. Role of ultrasound in manual detorsion for testicular torsion. J Clin Ultrasound. 2021;49:860–9. 34240428 10.1002/jcu.23039

[8] Hidaka AK Glina FPA Hayashi RM. Testicular decompression and tunica vaginalis flap in human acute testicular torsion: modified step-by-step technique description and preliminary outcomes. Einstein (Sao Paulo). 2023;21:eAO0220. 37585887 10.31744/einstein_journal/2023AO0220 PMC10421604

[9] Guo X Sun L Lei W Li S Guo H. Management of testicular torsion <360° in children: a single-center, retrospective study. J Int Med Res. 2020;48:300060519895861. 31891289 10.1177/0300060519895861 PMC7645356

[10] Waldert M Klatte T Schmidbauer J Remzi M Lackner J Marberger M. Color Doppler sonography reliably identifies testicular torsion in boys. Urology. 2010;75:1170–4. 19913882 10.1016/j.urology.2009.07.1298

[11] Yang C Song B Tan J Liu X Wei GH. Testicular torsion in children: a 20-year retrospective study in a single institution. ScientificWorldJournal. 2011;11:362–8. 21336452 10.1100/tsw.2011.39 PMC5720097

[12] Cuckow PM Frank JD. Torsion of the testis. BJU Int. 2000;86:349–53. 10930945 10.1046/j.1464-410x.2000.00106.x

[13] Drlík M Kočvara R. Torsion of spermatic cord in children: a review. J Pediatr Urol. 2013;9:259–66. 22763105 10.1016/j.jpurol.2012.05.016

[14] Yecies T Bandari J Schneck F Cannon G. Direction of rotation in testicular torsion and identification of predictors of testicular salvage. Urology. 2018;114:163–6. 29203186 10.1016/j.urology.2017.11.034

[15] Moore SL Chebbout R Cumberbatch M. Orchidopexy for testicular torsion: a systematic review of surgical technique. Eur Urol Focus. 2021;7:1493–503. 32863201 10.1016/j.euf.2020.07.006

[16] Bilagi P Sriprasad S Clarke JL Sellars ME Muir GH Sidhu PS. Clinical and ultrasound features of segmental testicular infarction: six-year experience from a single centre. Eur Radiol. 2007;17:2810–8. 17611760 10.1007/s00330-007-0674-2

[17] Bentley DF Ricchiuti DJ Nasrallah PF McMahon DR. Spermatic cord torsion with preserved testis perfusion: initial anatomical observations. J Urol. 2004;172(6 Pt 1):2373–6. 15538271 10.1097/01.ju.0000145527.08591.27

[18] Bowlin PR Gatti JM Murphy JP. Pediatric testicular torsion. Surg Clin North Am. 2017;97:161–72. 27894425 10.1016/j.suc.2016.08.012

[19] Karaguzel E Kadihasanoglu M Kutlu O. Mechanisms of testicular torsion and potential protective agents. Nat Rev Urol. 2014;11:391–9. 24934447 10.1038/nrurol.2014.135

